# Nonmuscle-invasive and Muscle-invasive Urinary Bladder Cancer: Image Quality and Clinical Value of Reduced Field-of-view Versus Conventional Single-shot Echo-planar Imaging DWI

**DOI:** 10.1097/MD.0000000000002951

**Published:** 2016-03-11

**Authors:** Yanchun Wang, Zhen Li, Xiaoyan Meng, Xuemei Hu, Yaqi Shen, John Morelli, Hui Lin, Zhongping Zhang, Daoyu Hu

**Affiliations:** From the Department of Radiology (YW, ZL, XM, XH, YS, DH), Tongji Hospital, Tongji Medical College, Huazhong University of Science and Technology, Wuhan, Hubei, China; St John's Medical Center (JM), Tulsa, OK; and Department of GE Healthcare (HL, ZZ), China.

## Abstract

This study compared the imaging quality, diagnostic accuracy, and apparent diffusion coefficient (ADC) values of reduced field-of-view (rFOV) diffusion-weighted imaging (DWI) and full field-of-view (fFOV) single-shot echo-planar imaging with regard to patients with nonmuscle-invasive or muscle-invasive bladder cancer.

Thirty-nine patients with 60 bladder tumors underwent rFOV and fFOV DWI in this internal review board-approved study. Pathologic and histologic grades were determined for all tumors. Two observers rated DWI image quality using a 4-point scale. Two radiologists who were blinded to the pathology findings reviewed 3 image sets (T2-weighted alone, T2-weighted plus fFOV DWI, and T2-weighted plus rFOV DWI) and assigned T stages and confidence levels for tumors of stage T2 or higher. The image quality scores for the 2 DWI sequences were assessed using the Wilcoxon signed-rank test. Differences in the diagnostic accuracy, sensitivity, and specificity for each image set were evaluated using the McNemar test. Differences in performance were analyzed by comparing the areas under the receiver-operating characteristic curves (ie, the *A*z values). A Mann–Whitney *U* test was used to compare the mean ADCs and the relationship between tumor stage and histologic grade.

Image quality scores were significantly higher for rFOV (mean = 3.62) than for fFOV DWI (2.98; *P* < 0.001). The pooled diagnostic accuracies were 57%, 70%, and 78% for the T2-weighted alone images, the T2-weighted plus fFOV DWI images, and the T2-weighted plus rFOV DWI images, respectively. The overall accuracy, specificity, and *A*z for diagnosing T2 or higher stages were significantly improved by adding rFOV DWI (*P* < 0.05). The mean ADC values of the muscle-invasive and G3 grade bladder cancers were significantly lower than those of the nonmuscle-invasive tumors and G1 grade cancers, regardless of DWI sequence (*P* < 0.01).

rFOV DWI is superior to fFOV DWI with respect to image quality and diagnostic accuracy. ADC values might be useful for distinguishing nonmuscle-invasive from muscle-invasive cancers, and G1 from G3 grade lesions.

## INTRODUCTION

Urinary bladder cancer is the most common malignant tumor of the urinary tract. The incidence rates of bladder cancer are 6% for men and 2% for women.^1^ The clinical treatment of bladder cancer depends on whether the muscularis propria of the urinary bladder is invaded (stage T2 or higher) or not (stage T1 or lower). Nonmuscle-invasive tumors are usually treated with transurethral resection (TUR) with or without supporting intravesicular chemotherapy or photodynamic treatment,^2^ whereas invasive tumors are treated with radical cystectomy, radiation therapy, chemotherapy, or a combination therapy.^3^ Therefore, the invasion of the bladder wall musculature is important to identify before treatment.

The value of diffusion-weighted imaging (DWI) with regard to distinguishing between nonmuscle-invasive and muscle-invasive tumors of the bladder has received much attention. DWI MRI shows a higher diagnostic accuracy for the staging of bladder tumors compared with T2-weighted images because of its favorable contrast resolution and ability to reflect molecular diffusion restriction in malignant tissue.^4–6^ Currently, full field-of-view (fFOV) single-shot echo-planar imaging (SS-EPI) is the standard sequence used for clinical DWI. However, the spatial resolution of this technique is restricted, and the technique is also limited by artifacts due to magnetic susceptibility.^7^ Artifacts and image distortions cause signal loss, which limits the ability to identify muscular invasion and potentially impairs the detection of small lesions, thereby reducing diagnostic accuracy.

Reduced field-of-view DWI (rFOV DWI) uses 2-dimensional radiofrequency (2DRF) pulses and a 180° refocusing pulse to reduce the FOV in the phase-encode direction. This sequence reduces the required readout duration for SS-EPI, not only reducing artifacts, but also resulting in greater image quality and higher-resolution DWI.^7^ This technique has been widely studied in other applications including the imaging of the spine,^7,8^ the brain,^9^ the breast,^10,11^ the thyroid,^12^ and the pancreas.^13^ To the best of our knowledge, however, no previous reports have described urinary bladder cancer T staging using rFOV DWI.

This study sought to compare the image quality, diagnostic accuracy, and ADC values of fFOV DWI and rFOV DWI with regard to patients with nonmuscle-invasive and those with muscle-invasive urinary bladder tumors to determine whether the latter can reliably depict muscular invasion.

## METHODS

### Patients

The Ethics Committee of Tongji Hospital of Huazhong University of Science and Technology approved this study, and all patients provided written informed consent before their inclusion. Between January 2014 and December 2014, 59 consecutive adult patients with clinically suspected bladder cancers, presenting with gross hematuria, bladder malignancy identified on outpatient cystoscopy, or both, were included in this study. The exclusion criteria were contraindications to MRI or refusal to undergo MRI.

**FIGURE 1:**
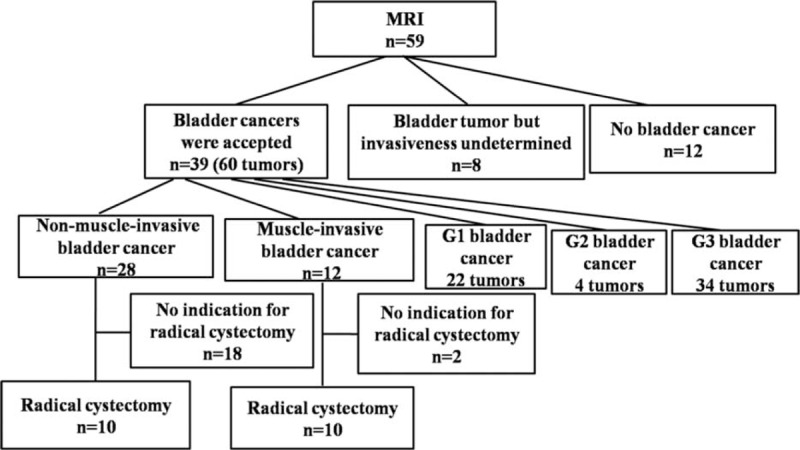
Flow diagram demonstrating patient and tumor characteristics.

All 59 patients underwent MRI, and all patients eventually underwent cystoscopy. Twelve patients were ultimately found to not have bladder cancer and were excluded from the study. The remaining 47 patients underwent further treatment, TUR, or radical cystectomy. MRI scans of these patients were conducted 1 to 31 days (mean = 5 days) before TUR or radical cystectomy treatment. Eight patients were excluded because the invasive nature of their cancers was not histologically confirmed (2 cases of adenocarcinoma, 1 case of prostate cancer metastasis, 1 case of colon cancer metastasis, and 1 case of bladder papilloma; 5 additional patients declined surgery). Thus, 39 patients were examined in this study. Patients with muscle-invasive bladder cancer and those with high-risk nonmuscle-invasive disease not cystoscopically controllable, who were in otherwise good physical condition without nodal or distant metastases, were considered for radical cystectomy treatment (Figure 1).

Pathologic stage was confirmed in 39 patients (4 women and 35 men; age range = 37–81 years; median age = 62 years). The specimens were stained with hematoxylin and eosin stain for histopathologic evaluation. Nine patients had multiple lesions (21 additional tumors); thus, 60 bladder cancers were analyzed.

### MRI Technique

To moderately distend the urinary bladder, the patients were prohibited from urinating for at least 1 hour before examination.

All scans were performed using a 3.0T MRI scanner (Discovery 750, GE Medical System, Milwaukee, WI) in the supine position. A 32-channel torso phased-array coil was used to image the bladder.

Before DW MRI, sagittal T2-weighted images and axial T1-weighted images were obtained for all patients using a fast spin-echo sequence (for sagittal images: repetition time [TR]/echo time [TE] = 4902/130 ms, field of view [FOV] = 240 mm × 240 mm, matrix size = 320 × 320, section thickness = 4 mm, intersection gap = 0.4 mm, echo train length = 24, readout bandwidth = 62.5 kHz, 22 sections in 2 minutes and 15 seconds; for axial images: 528/minimum, FOV = 340 mm × 340 mm, matrix size = 288 × 256, section thickness = 4 mm, intersection gap = 1 mm, echo train length = 4, bandwidth = 50 kHz, 28 sections in 1 minute and 58 seconds). T2-weighted images in the axial orientation were obtained for all of the patients using a fat-suppressed fast recovery fast spin echo sequence (TR/TE = 3708/68 ms, FOV = 340 mm × 340 mm, matrix size = 320 × 256, section thickness = 4 mm, intersection gap = 1 mm, echo train length = 16, readout bandwidth = 62.5 kHz, 28 slices in 2 minutes and 32 seconds).

The scanning parameters of the axial SS-EPI DWI (fFOV DWI) were as follows: TR = 4000 ms, shortest TE, FOV = 340 mm × 340 mm, matrix = 160 × 128, slice thickness = 4 mm, gap = 1 mm, bandwidth = 250 kHz, NEX = 12, and *b* = 0 and 800 s/mm^2^ (DW gradients applied in three orthogonal directions), 28 slices in 2 minutes and 32 seconds. The entire bladder and occasionally its above organization were covered using this method. The pixel size using fFOV is approximately 5.64.

Reduced field-of-view SS-EPI DWI was also obtained in the axial plane. The rFOV DWI scanning parameters were as follows: TR = 4000 ms, shortest TE, FOV = 240 mm × 96 mm, matrix = 128 × 96, slice thickness = 4 mm, gap = 1 mm, bandwidth = 250 kHz, NEX = 12, *b* = 0 and 800 s/mm^2^ (DW gradients applied in three orthogonal directions), 16 slices in 2 minutes and 32 seconds. The entire bladder was covered as soon as possible using this method. Occasionally, the bladder was too full; thus, we might have missed some of the normal bladder wall. The pixel size using rFOV is approximately 1.88.

### Imaging Quality Assessment

Both rFOV DWI and fFOV DWI MRIs were made anonymous and reviewed on a picture archiving and communication system (PACS) workstation (AGFA Impax). Two independent observers, 1 radiologist (YW) with 5 years of experience in abdominal radiology (reader 1) and another radiologist (XM) with 3 years of experience in abdominal radiology (reader 2), rated the image (*b* = 800 s/mm^2^) quality for both the fFOV DWI and rFOV DWI and specified a score using a 4-point scale,^13^ where 1 = poor image quality, considered nondiagnostic; 2 = fair image quality, somewhat impairing diagnostic quality; 3 = good image quality, not impairing diagnostic quality; 4 = excellent image quality. This scale was used to reflect overall image quality, including the salience of the bladder tumor (s) and the degree of artifact present.

### Identifying Muscular Invasion

Images were analyzed using the PACS workstation and were independently reviewed by 2 radiologists (ZL and DH, with 12 and 29 years of abdominal imaging experience, respectively) who were blinded to the biopsy/pathology results. Each radiologist reviewed 3 image sets: the axial T2-weighted images alone, the axial T2-weighted plus axial fFOV DWI images, and the axial T2-weighted plus axial rFOV DWI images. First, the T2-weighted images alone and the T2-weighted plus fFOV DWI images were analyzed (in that order); then, the T2-weighted plus rFOV DWI images were analyzed after 3 weeks. The patients’ data were put in a random order and evaluated by the 2 reviewers. The 3-week interval was meant to minimize the recall of individual cases. The readers were asked to distinguish nonmuscle-invasive (stage T1 or lower) from muscle-invasive (stage T2 or higher) tumors. Differences in assessment were resolved via consensus.

The following staging criteria were implemented, which are similar to those used in previous studies for T2WI^14–16^ and DWI.^4^ On T2WI, the normal bladder wall presented a complete low signal intensity (SI) line. When the low SI line was present and intact, the bladder cancers were considered as stage T1 or lower; when the low SI line was disrupted and invaded by the tumor, the bladder cancers were considered as stage T2 or higher.

On DW images, bladder cancers demonstrate high SI. In accordance with a prior report,^4^ a thin or flat area of increased SI corresponding to tumor with thickened submucosa or a low signal submucosal stalk was considered to indicate a T1 stage tumor, whereas a tumor with a smooth margin but without a stalk was considered to indicate a stage T2 tumor. Extent into the perivesicular fat (stage T3) and extent into the adjacent organs (stage T4) were also considered.

In addition, the 2 radiologists used a 4-point scale to determine whether the bladder tumor was stage T2 or higher and gave a confidence level,^6^ where 1 = definitely absent; 2 = possibly absent; 3 = possibly present; and 4 = definitely present. And then we calculated the sensitivity and specificity of the MR sequences, which are similar to those used in previous study.^6^ We calculated the sensitivity of bladder tumors at stage T2 or higher based on the patients’ number whose tumors had a 3 or 4 confidence level by radiologist consensus and the number of patients with tumors pathologic stage T2 or higher. And, the specificity was calculated based on the patients’ number whose tumors had a 1 or 2 confidence level and the number of patients whose tumors were pathologic stage T1 or lower.

### ADC Values of Bladder Cancers

All of the ADC values were calculated on a workstation with a standard software package (ADW4.5; GE Medical Systems). For tumors of sufficient size (>5 mm), the ADC value of a tumor was measured by carefully drawing a region of interest (ROI) around the largest area of tumor identified on both *b* = 0 s/mm^2^ rFOV DWI and fFOV DWI images. This step was performed 3 times for each patient by a single observer (YW with 5 years of experience in MRI). The average of 3 measurements was taken as the average ADC value.

### Statistical Analyses

The results of the image quality scores for the 2 DWI sequences were assessed using the Wilcoxon signed-rank test. Inter-reader correlation was estimated using Pearson correlation coefficient with regard to the image quality of the rFOV DWI and fFOV DWI. The diagnostic accuracy of the T staging with MRI compared with the pathologic staging was assessed on a stage-by-stage basis. Differences in the sensitivities, specificities, and diagnostic accuracies for each image set were evaluated using the McNemar test. Interobserver agreement was calculated using kappa statistics. A kappa value of less than 0.20 was considered as poor agreement, whereas 0.21 to 0.40 was considered as fair, 0.41 to 0.60 was considered as moderate, 0.61 to 0.80 was considered as good, and 0.81 to 1.00 was considered as excellent agreement. The Mann–Whitney *U* test was used to compare the mean ADCs in the relationship between the tumor stage and histologic grade. All analyses were performed using SPSS for Windows, version 19.0 (IBM, Armonk, NY).

Receiver-operating characteristic (ROC) curves were fit to the radiologists’ confidence ratings, and observer performance for each sequence was estimated by calculating the area under the ROC curve (*Az*). Medcalc (version 12.7.0.0; MedCalcSoftware, Mariakerke, Belgium) was employed to draw the ROC curve and estimate the differences between the *Az* values.

*P* values less than 0.05 were considered as significant.

## RESULTS

### Tumor Characteristics

Of the 9 patients with multiple lesions, 1 had 3 lesions; 1 tumor was muscle-invasive, and the other 2 were not.

Nonmuscle-invasive bladder cancers (Tis and T1) were diagnosed in 28 patients (in 80% [48 of 60] of the tumors), and muscle-invasive bladder cancers (≥T2) were present in 12 of 60 patients (20%; T2 in 12% [7 of 60], T3 in 3% [2 of 60], T4 in 5% [3 of 60]). Ten of the 28 patients with nonmuscle-invasive cancer and 10 of the 12 patients with muscle-invasive cancer underwent radical cystectomy (Figure 1).

A total of 59 transitional cell carcinomas and 1 case of bladder adenocarcinoma were identified via histologic diagnoses. The histologic grade of the 60 tumors was as follows: G1 in 22 tumors (36.7%), G2 in 4 (6.7%), and G3 in 34 (56.7%; Figure 1).

Two patients’ tumors were very small, and the measurements were discarded. One case was in stage T1, and the other case was in stage T4.

### Comparison of Image Quality and the Interobserver Variability of Image Quality

The image quality scores assessed by the 2 readers for the rFOV DWI and fFOV DWI scans are shown in Table 1.

**TABLE 1:**
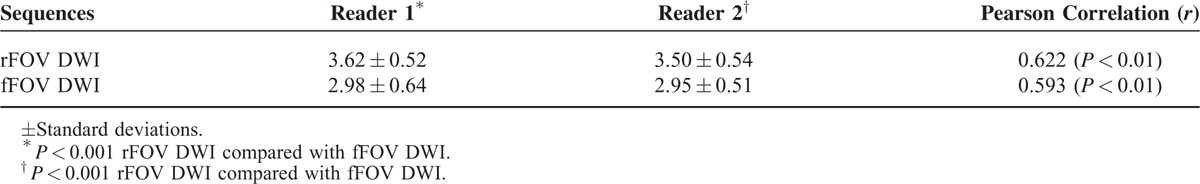
Subject Image Quality Scores (Mean ± SD) of rFOV DWI and fFOV DWI (Reader 1 and Reader 2, Interobserver Comparison)

**FIGURE 2:**
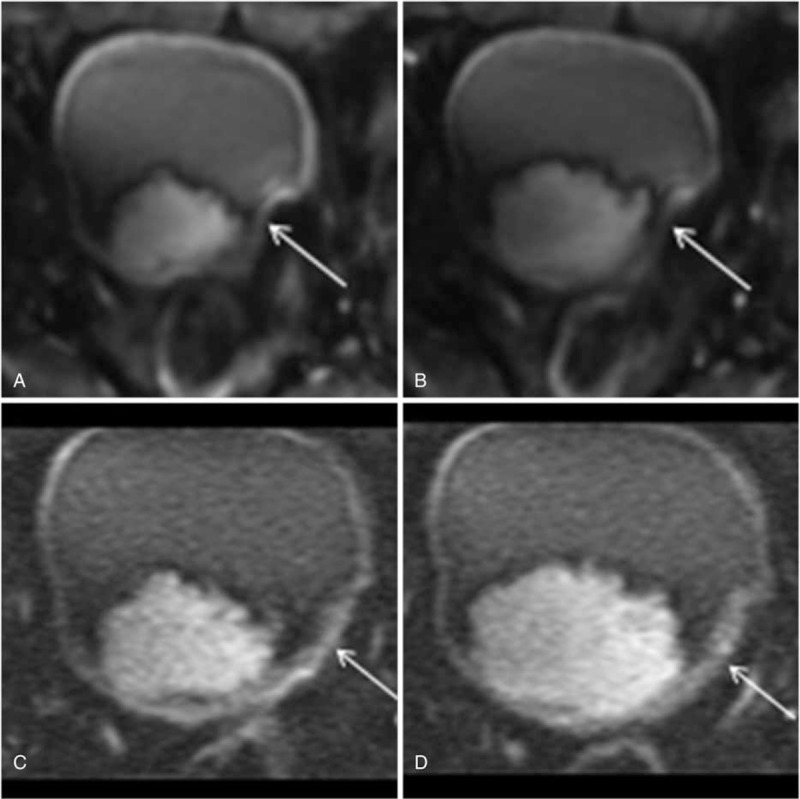
A 64-year-old man with stage pT1 papillary urothelial carcinoma. A, B, The axial fFOV DW MRI shows a severe magnetic susceptibility artifact at the junction of the bladder and rectum (arrow). C, D, The axial rFOV DW MRI demonstrates a significantly reduced artifact in this location (arrow). DW = diffusion-weighted, fFOV = full field of view, MRI = magnetic resonance imaging, rFOV = reduced field of view.

The subjective image quality scores were significantly higher for rFOV DWI at *b* value 800 s/mm^2^ based on observations made by both readers (Figure 2). The image quality scores for rFOV DWI at *b* value of 800 s/mm^2^ were 3.62 ± 0.52 and 3.50 ± 0.54 for readers 1 and 2, respectively. The image quality scores for fFOV DWI at *b* value 800 s/mm^2^ were 2.98 ± 0.64 and 2.95 ± 0.51 for readers 1 and 2, respectively. A high inter-rater correlation was found for the rFOV DWI and fFOV DWI image quality ratings between readers 1 and 2.

### MRI for Tumor Staging

**FIGURE 3:**
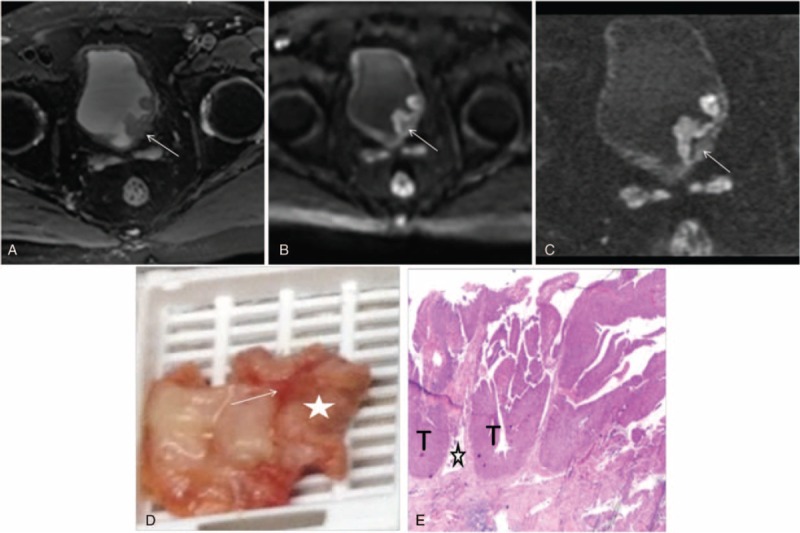
A 69-year-old man with multiple stages of pT1 papillary urothelial carcinoma. A, The T2-weighted imaging shows tumor tissue with intermediate SI within the posterior urinary bladder. The SI of the muscle layer at the base of the tumor is slightly elevated (arrow). B, The axial fFOV DW MRI shows a C-shaped high-SI area with a low-SI stalk connected to the posterior side of the bladder wall (arrow). C, The axial rFOV DW MRI shows the tumor and stalk more clearly (arrow). D, The corresponding specimen obtained on radical cystectomy shows a papillary tumor tissue (star) with a submucosal stalk (arrow). E, The photomicrograph of the specimen shows papillary cancer (T) with a submucosal stalk (star); hematoxylin and eosin stain; original magnification, ×100. DW = diffusion-weighted, fFOV = full field of view, MRI = magnetic resonance imaging, rFOV = reduced field of view, SI = signal intensity.

**FIGURE 4:**
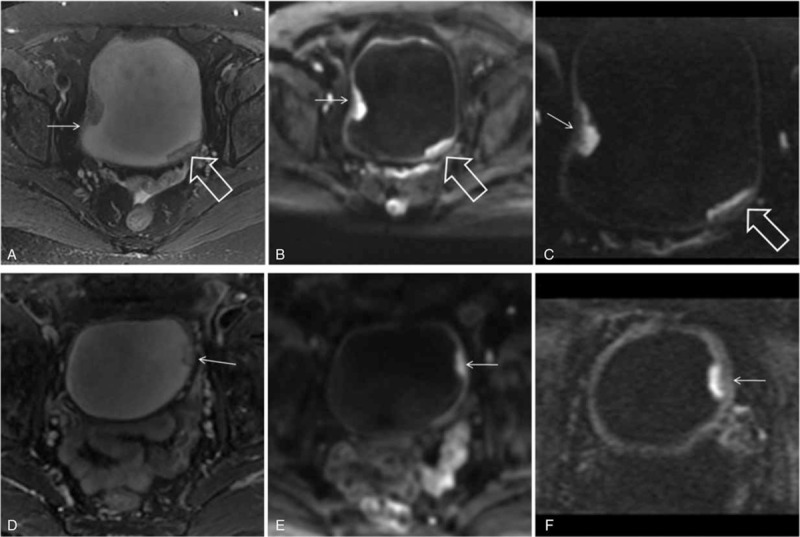
A 51-year-old man with multiple stages of pT1 urothelial carcinoma (A–C) and a 70-year-old man with stages of pT1 papillary urothelial carcinoma (D–F). A, D, The axial T2-weighted MRI shows elongated oval masses along the right bladder wall (arrow) and the left lateral wall (arrow and open arrow). The SI of the muscle layer at the base of the 3 tumors is slightly elevated, and the low SI line was disrupted. B, E, The axial fFOV DW MRI shows 2 high SI tumors without a submucosal stalk, but with a smooth tumor margin and submucosal thickening (arrow and open arrow). C, F, The axial rFOV DW MRI shows the 2 high SI tumors with a thickened submucosa (arrow and open arrow). DW = diffusion-weighted, fFOV = full field of view, MRI = magnetic resonance imaging, rFOV = reduced field of view.

**FIGURE 5:**
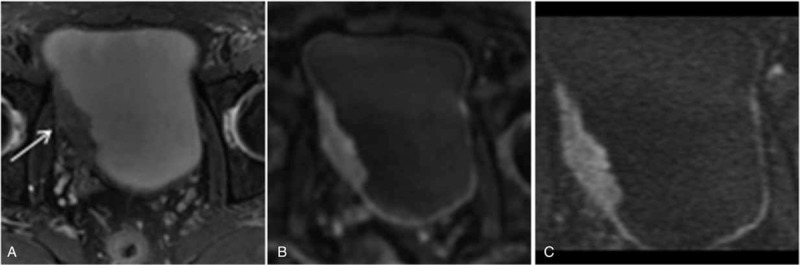
A 51-year-old man with stages of pT2b urothelial carcinoma. A, The axial T2-weighted MRI shows an ovoid mass along the right urinary bladder wall. The tumor extends into the perivesical fat with an irregular contour (arrow). B, The axial fFOV DW MRI shows a bulging tumor with a smooth margin on the right wall of the bladder. C, The axial rFOV DW MRI shows a bulging tumor with a smooth margin on right wall of the bladder more clearly. DW = diffusion-weighted, fFOV = full field of view, MRI = magnetic resonance imaging, rFOV = reduced field of view.

The interobserver agreement of each interpretation and the overall staging accuracy are summarized in Table 2. The interobserver agreement was excellent for the T2-weighted alone images and good for the T2-weighted plus fFOV DWI and T2-weighted plus rFOV DWI images. In 42 of 60 cases (70%, *P* = 0.08), the tumor was correctly staged with T2-weighted images plus fFOV DWI compared with 47 of 60 cases (78%, *P* = 0.001) using T2-weighted images plus rFOV DWI. These results were significantly better than those obtained using the T2-weighted images alone (34 of 60 [57%]; Figures 3 and 4). The diagnostic accuracy of T2-weighted plus rFOV DWI was significantly better than that associated with the results obtained using T2-weighted plus fFOV DWI (*P* = 0.025; Figure 5).

**TABLE 2:**
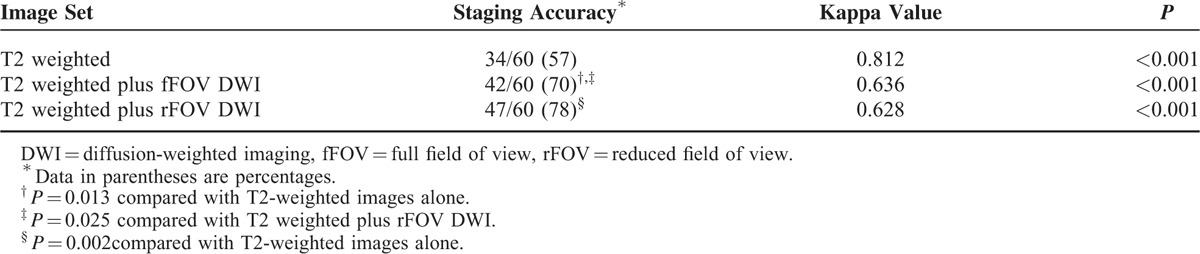
Overall Accuracy and Interobserver Agreement

The sensitivities, specificities, accuracies, and *Az* values for the detection of urinary bladder tumors at stage T2 or higher are summarized in Table 3. The sensitivity was lower with both diffusion sequences (both 75%) than the T2-weighted imaging alone (92%) (*P* = 0.625). The specificities obtained using T2-weighted plus rFOV DWI were significantly higher than those obtained using the T2-weighted images alone (*P* = 0.004) or the T2-weighted plus fFOV DWI images (*P* = 0.008). The accuracies obtained using the T2-weighted plus rFOV DWI images were also higher than those obtained using the T2-weighted images alone (*P* = 0.029) or the T2-weighted plus fFOV DWI images (*P* = 0.008). The *Az* values for detecting stage T2 or higher tumors were better using the T2-weighted plus rFOV DWI images (0.826) than either the T2-weighted plus fFOV DWI (0.771; *P* = 0.0049) or T2-weighted alone images (0.781; *P* = 0.6522).

**TABLE 3:**
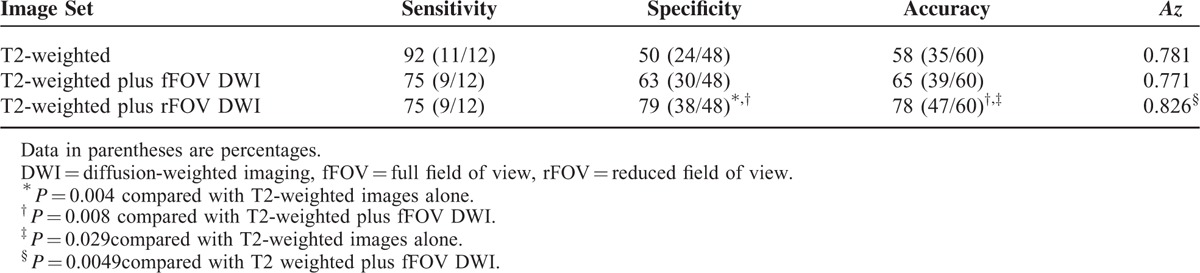
Sensitivity, Specificity, Accuracy, and Area Under the Receiver-operating Characteristic Curve (*Az*) for Tumors of Stage T2 or Greater

### The Association Between the ADC Value and Tumor Stage

The fFOV DWI and rFOV DWI ADCs of nonmuscle-invasive tumors (stage T1 or lower) and muscle-invasive tumors (stage T2 or higher) are shown in Table 4.

**TABLE 4:**
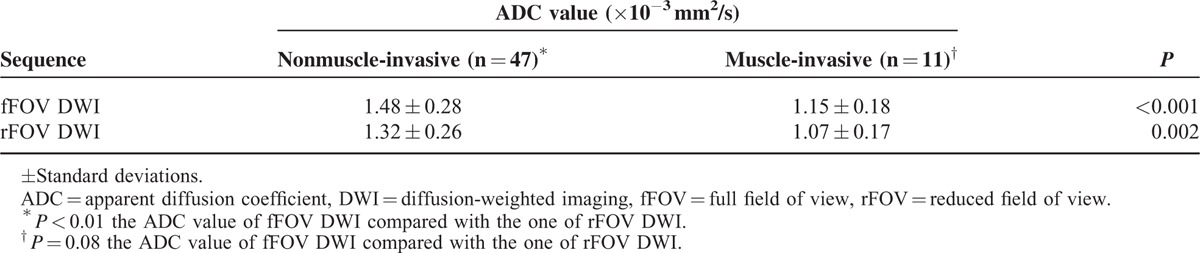
Mean ADC Values for Nonmuscle Invasive and Muscle-invasive Bladder Cancer

The mean ADCs of nonmuscle-invasive and muscle-invasive tumors for fFOV DWI were 1.48 ± 0.28 × 10^−3^ and 1.15 ± 0.18 × 10^−3^ mm^2^/s, respectively. The mean ADCs of nonmuscle-invasive and muscle-invasive tumors on rFOV DWI were 1.32 ± 0.26 × 10^−3^ and 1.07 ± 0.17 × 10^−3^ mm^2^/s, respectively. The differences in ADC values were significant between nonmuscle-invasive tumors and muscle-invasive tumors on fFOV DWI and rFOV DWI (*P* < 0.001 and 0.002, respectively).

The mean ADC values between fFOV DWI and rFOV DWI for nonmuscle-invasive tumors significantly differed (*P* < 0.001).

The mean ADC values between fFOV DWI and rFOV DWI for muscle-invasive tumors did not significantly differ (*P* = 0.080)

### The Association Between the ADC Values and Histologic Grade

Four tumors had a histologic grade of G2. Because of this small number, these tumors were excluded from comparisons. Tumors of histologic grades G1 and G3 were analyzed. The mean ADCs corresponding to histologic grades G1 and G3 are summarized in Table 5.

**TABLE 5:**
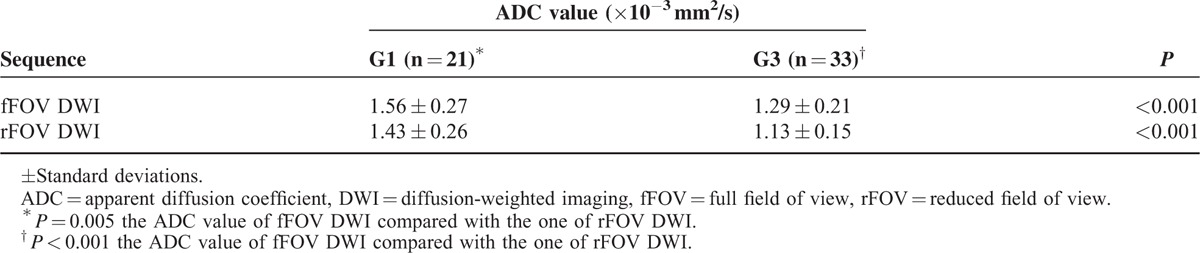
Mean ADC Values for Different Histological Grades of Bladder Cancer

The mean ADCs of the G1 and G3 tumors for fFOV DWI were 1.56 ± 0.27 × 10^−3^ and 1.29 ± 0.21 × 10^−3^ mm^2^/s, respectively.

The mean ADCs of the G1 and G3 tumors for rFOV DWI were 1.43 ± 0.26 × 10^−3^ and 1.13 ± 0.15 × 10^−3^ mm^2^/s, respectively. The ADC significantly differed between the G1 and G3 tumors on fFOV DWI and rFOV DWI (*P* *<* 0.001, 0.001).

The mean ADC values between fFOV DWI and rFOV DWI for both G1 (*P* = 0.005) and G3 (*P* < 0.001) tumors significantly differed.

## DISCUSSION

Several imaging methods such as computed tomography (CT), T2-weighted MRI, dynamic MRI, and DW MRI are useful in determining the clinical stage of urinary bladder cancer.^4–6,17–20^ The rFOV DWI technique produces fewer artifacts and less geometric distortion than traditional fFOV DWI with regard to several clinical applications.^7^ Our study sought to compare the image quality, diagnostic accuracy, and ADC values of fFOV DWI and rFOV DWI with regard to patients with nonmuscle-invasive and those with muscle-invasive bladder tumors.

In the present study, the image quality of rFOV DWI was significantly greater than that of fFOV DWI based on the observations of readers 1 and 2, who showed excellent agreement. This result is consistent with those of previous studies.^9–11,13,21^ For example, Singer et al^10^ and Dong et al^11^ used rFOV DWI and SS-EPI DWI to study breast cancer, showing that rFOV DWI enabled imaging with increased spatial resolution and decreased susceptibility to artifacts and fewer chemical shift artifacts. Ma et al^13^ compared rFOV DWI and SS-EPI DWI with regard to the pancreas and found that the former reduced blurring artifact and provided higher resolution imaging than the latter. These results are attributable to several factors. rFOV DWI reduces the number of phase-encoding (PE) lines via the reduction of the FOV in the PE direction, thereby also reducing TE time. The bandwidth in the direction of the PE is therefore increased, which reduces anatomic distortions and artifacts. Although spectral fat saturation is applied using both sequences, the increased bandwidth in the PE dimension also reduces residual fat misregistration due to chemical shift,^11^ which might be helpful for depicting small soft tissue structures. To determine whether bladder cancer invades or spares the muscularis propria of the bladder wall, several signs are relied upon including the presence of a low SI submucosal stalk or a thickened submucosa. rFOV DWI more clearly depicts these findings and therefore improves T-staging accuracy compared with fFOV DWI. In the present study, the overall accuracy for diagnosing tumor stage using the T2-weighted images plus fFOV DWI was significantly greater than that achieved using the T2-weighted alone images. The interobserver agreement ranged from good to excellent for all sequences in agreement with previous reports.^4–6^ The overall staging accuracy of T2-weighted imaging is 40% to 67% according to reports published over the past 10 years.^4–6,19^ Tekes et al^19^ reported that 81% of bladder tumors show SI values similar to muscle on T2-weighted imaging, therefore resulting in a tendency to overestimate the T stage. The overall accuracy of T2-weighted MRI in staging bladder cancer in our study was 57%, which is similar to that observed by Tekes et al. The overall accuracy of T2-weighted plus fFOV DWI in our study was 70%, which was slightly less than that reported in previous studies.^4^ The accuracy in our study might have been diminished because some patients underwent biopsy before MRI, potentially altering their tumor SI, leading to an underestimation of tumor stage.^22–24^ The overall accuracy of T2-weighted plus rFOV DWI in our study was 78%, which was significantly higher than that of T2-weighted plus fFOV DWI (70%). The specificity and *A*z for diagnosing T2 or higher stages were significantly improved by adding rFOV DWI. This improvement is attributable to the decreased susceptibility artifacts and the increased image quality using rFOV DWI, specifically with respect to the improved visualization of the primary tumor and its associated low SI stalk or submucosal thickening, and the improved visualization of the muscular layer.

Many DWI studies evaluating urinary bladder tumors have been conducted recently. Matsuki et al^25^ showed that the ADC values in tumors were significantly lower than those in the surrounding normal tissue. Avcu et al^26^ found that the ADC values in carcinomas were significantly lower than those in benign tumors. In another study of 43 patients with bladder tumors, El-Assmy et al^27^ found ADC values that were significantly lower in bladder carcinomas than in surrounding tissues. In our study, the ADC values of muscle-invasive tumors were significantly lower than those of the tumors that did not invade the muscularis propria, regardless of whether the fFOV or rFOV DWI technique was applied. This result is consistent with those of other studies in the literature. In addition, the mean ADC values of the G3 tumors were significantly lower than those of the G1 tumors using both fFOV DWI and rFOV DWI. This result is also consistent with previous studies.^4,26^ The above results might be explained by the fact that more aggressive malignant lesions have a denser cellular structure, which leads to lower ADC values. Therefore, ADC values might partially help distinguish the tumor and histologic grades of bladder cancer. The ADC values obtained with rFOV DWI were also lower than those obtained with fFOV DWI, regardless of pathological stage or histologic grade. This finding might be accounted for by the reduction in the partial volume effects between the tumor and normal tissue using rFOV DWI, which results in lower overall and potentially more accurate ADC values.^10^

The present study has numerous limitations. The sample size was relatively small; only 39 patients were enrolled. In addition, the distribution of T stages was uneven, with a large number of nonmuscle-invasive tumors and a smaller number of invasive tumors. Thus, studies with a larger sample sizes are warranted to more fully define the role of DWI (especially rFOV DWI) in the T staging of bladder tumors. The histologic grade distribution was also nonuniform with many G1 and G3 tumors; however, only 4 patients had G2 grade tumors. Thus, G2 tumors were excluded from analysis, and comparisons to G1 and G3 lesions were not performed. The other limitation was that some patients underwent biopsy before MRI, which might affect the staging of bladder cancer. Finally, another unmet need in bladder cancer staging is lymph node assessment, and DWI is a subtle imaging method for lymph nodes. We did not assess lymph nodes in this study. However, future research is needed to estimate whether DWI is useful in this regard.

In conclusion, rFOV DWI can depict the urinary bladder with fewer artifacts and less anatomic distortion than fFOV DWI. The combination of T2-weighted and rFOV DWI resulted in the greatest diagnostic accuracy, particularly with respect to determining muscle-invasive versus nonmuscle-invasive tumors. The presence of a low tumor ADC value also suggests that a tumor is more likely to be muscle-invasive or of higher grade (ie, G3). rFOV DWI might be a superior alternative to determine urinary bladder tumor T stage over fFOV DWI.
